# Personalized life expectancy and treatment benefit index of antiretroviral therapy

**DOI:** 10.1186/s12976-016-0047-0

**Published:** 2017-01-18

**Authors:** Yanni Xiao, Xiaodan Sun, Sanyi Tang, Yicang Zhou, Zhihang Peng, Jianhong Wu, Ning Wang

**Affiliations:** 1 Department of Applied Mathematics, Xi’an Jiaotong University, Xianning West Road, Xi’an, 710049 China; 2 College of Mathematics and Information Science, Shaanxi Normal University, West Chang’an Avenue, Xi’an, 710119 China; 3 School of Public Health, Nanjing Medical University, Nanjing, 210029 China; 4 Laboratory for Industrial and Applied Mathematics, Centre for Disease Modelling, York Institute for Health Research, York University, Toronto, M3J 1P3 Canada; 5 National Center for AIDS/STD Prevention and Control, Chinese Center for Disease Control and Prevention, 155 Changbai Road, Beijing, 102206 China

**Keywords:** Viral dynamic model, HIV, Antiretroviral therapy, Life expectancy, Treatment benefit index

## Abstract

**Background:**

The progression of Human Immunodeficiency Virus (HIV) within host includes typical stages and the Antiretroviral Therapy (ART) is shown to be effective in slowing down this progression. There are great challenges in describing the entire HIV disease progression and evaluating comprehensive effects of ART on life expectancy for HIV infected individuals on ART.

**Methods:**

We develop a novel summative treatment benefit index (TBI), based on an HIV viral dynamics model and linking the infection and viral production rates to the Weibull function. This index summarizes the integrated effect of ART on the life expectancy (LE) of a patient, and more importantly, can be reconstructed from the individual clinic data.

**Results:**

The proposed model, faithfully mimicking the entire HIV disease progression, enables us to predict life expectancy and trace back the timing of infection. We fit the model to the longitudinal data in a cohort study in China to reconstruct the treatment benefit index, and we describe the dependence of individual life expectancy on key ART treatment specifics including the timing of ART initiation, timing of emergence of drug resistant virus variants and ART adherence.

**Conclusions:**

We show that combining model predictions with monitored CD4 counts and viral loads can provide critical information about the disease progression, to assist the design of ART regimen for maximizing the treatment benefits.

## Background

Human immunodeficiency virus (HIV), the pathogen causing acquired immune deficiency syndrome (AIDS), exhibits highly complex interaction with human immune system [1, 2]. HIV infection typically results in a vast virus replication during the acute infection phase that is followed by a chronic phase where the viral load approaches a much lower quasi-steady state, and then followed by a sharp and sudden rise of viral loads when the immune system collapses [3–7]. Typical stages of HIV infection are well documented [6, 8], and the antiretroviral therapy (ART) is shown to be effective in slowing down the progression to AIDS and improving the life quality of HIV patients [9–11]. Most existing models however failed to describe the entire HIV disease progression trajectory partly, especially they could not model the significant increase of viral loads after the development of AIDS.

There has been substantial progress in modelling antiretroviral intervention, with particular success in predicting long-term viral dynamics [12–17]. A challenge in describing the entire HIV disease progression trajectory arises from the temporal variability of the infection rate and the viral reproduction rate [13, 16, 18]. One purpose of this study is to propose a novel viral dynamic model which can describe a typical disease progression including acute infection, chronic latency and AIDS stage on the basis of the classic viral dynamic model frame [8, 19–21]. We then show that parametrizing the infection rate and viral reproduction rate through three key parameters in the Weibull function [22, 23] permits us to extend the classical viral dynamics model in such a way that accurate description of the viral dynamics during the entire HIV disease progression within a host is possible. We also demonstrate, using a longitudinal cohort study in China, how parameters of the relevant Weibul functions can be estimated by fitting the viral dynamics model prediction to patient data, and how these parameterized Weibul functions in combination with the viral dynamics model yields important information about the comprehensive effects of ART on the life expectancy (LE).

Estimating the LE is important to inform the patients of their prognosis at the individual level, and to predict the future demographic and socioeconomic impact of HIV/AIDS at the population level. Several studies have investigated the prolonged LE of patients due to ART in high-income countries or resource-constrained settings at the population level [24–28], using observed mortality rates in various cohort studies. There are many challenges in determining the timing of infection, predicting the LE of HIV infected individuals and quantifying the comprehensive effects of ART on life expectancy. In the study here, based on parametrized temporal variability of infection rate and viral reproduction rate through the Weibul function which are incorporated in the classical viral dynamics model, our another purpose is to establish a predictive formula at the individual level for the LE of patients receiving ART, and to simulate how this individual LE is related to ART treatment specifics such as drug efficacy, sensitivity, adherence, treatment starting time and the time of emergence of drug resistant virus variants.

## Methods

**The model** Let *T*(*t*),*T* ^∗^(*t*) and *V*(*t*) be the concentrations of uninfected target CD4 T cells, productively infected cells, and free virus at time, respectively. We adopt the classic HIV viral dynamics model [19, 21], but include the temporal variability of infection rate and viral production rate in order to provide a faithful account of the entire disease progression of within an HIV patient. Namely, we have

1 $$ \left\{\begin{array}{l} \frac{dT}{dt}=s-d T-k(t) VT,\\ \frac{dT^{*}}{dt}=k(t) VT-\delta T^{*},\\ \frac{dV}{dt}=\lambda(t) T^{*}-c V,\\ \end{array}\right.  $$

where *s* is the rate of recruitment of uninfected cells, *d* and *δ* are the death rates of uninfected cells and infected cells respectively, *c* is the rate of clearance. We propose to link the temporally varying infection rate *k*(*t*) and viral production rate *λ*(*t*), in the absence of ART, to the Weibull function

2 $$ W\left(t, T_{m}, \beta, \alpha\right)=1-\exp\left[-\left(\frac{T_{m}-t}{\beta}\right)^{\alpha}\right], \ t<T_{m},  $$

with respective location parameter (*T*_*m*_), the shape parameter (*α*), and the scale parameter (*β*). The Weibull function is characterized by shape, scale and location parameters. The location parameter determines the maximum life span of the patient since infection. Using this local parameter, we can define and calculate a summative index, the treatment benefit index (TBI), as the difference of the LEs of a patient with and without ART, to measure the overall benefit of ART treatment in terms of the life year gained. Specifically, we have

3 $$ k(t)=\frac{k}{W\left(T_{m}, \beta_{k},\alpha_{k}\right)}=\frac{k}{1-\exp\left[-\left(\frac{T_{m}-t}{\beta_{k}}\right)^{\alpha_{k}}\right]}, \ t<T_{m},  $$

and

4 $$ \lambda(t)=\frac{\lambda}{W\left(T_{m}, \beta_{\lambda}, \alpha_{\lambda}\right)}=\frac{\lambda}{1-\exp\left[-\left(\frac{T_{m}-t}{\beta_{\lambda}}\right)^{\alpha_{\lambda}}\right]}, \ t<T_{m}.  $$

with normalized constants *k* and *λ*. Definitions of variables and parameters as well as the baseline parameter values are listed in Table 1. It is interesting to note that the viral loads within-host behave like the ‘bathtub curve’, which shows three stages over the life time and hence is very well depicted by the proposed mixed Weibull function.

**Table 1:** Definitions of the parameters used in the model

Variables	Definitions	Initial	Reference
*T*	Uninfected CD 4^+^ cell population size	1200 *μ* *l* ^−1^	
*T* ^∗^	Infected CD 4^+^ helper cell population size	0	
*V*_*I*_	HIV population size	100 *μ* *l* ^−1^	
*τ*	Prolonged LE	interim variable	
Parameters		baseline values [ranges]	
*s*	Rate of supply of CD 4^+^ T cell from precursors	15 *μ* *l* ^−1^ day ^−1^	[21]
*d*	Death rate of uninfected CD 4^+^ T cells	0.02 day ^−1^	[21]
$k(\bar {k})$	Infection rate per virion	2.1818×10^−7^ *μ* *l* ^−1^ day ^−1^(*lk*)	[21]
*δ*	Death rate of infected CD 4^+^ T cells	0.35 day ^−1^[0.2 0.6]	[21]
$\lambda (\bar {\lambda })$	Number of free virus produced by lysing a CD 4^+^ T cell	3928.6 *μ* *l* ^−1^ day ^−1^(*l* *λ*)	[21]
*c*	Death or clearance rate of free virus	2.4 day ^−1^ [1.5 3.5]	[21]
$\beta _{k}(\bar {\beta }_{k})$	Scale parameter of Weibull function	1500(225) [50-2000]	see text
$\beta _{\lambda }(\bar {\beta }_{\lambda })$	Scale parameter of Weibull function	200(200) [10-500]	see text
$\alpha _{k}(\bar {\alpha }_{k})$	Shape parameter of Weibull function	1.1(1.1) [0.2-2]	see text
$\alpha _{\lambda }(\bar {\alpha }_{\lambda })$	Shape parameter of Weibull function	0.04(0.04) [0.005-0.2]	see text
*T*_*m*_	LE since infection without therapy	11 [5,16] ×365 days	[34]
$\eta (\bar {\eta })$	Drug efficacy of combination therapy	0.95 [0.5 1] (*q* *η*)	[21]
$\tau _{50}(\bar {\tau }_{50})$	Drug sensitivity of combination therapy	30 [20, 100] ×365 days (*p* *τ*_50_)	see text
*τ*_*m*_	Maximum LE after infection	(*T*_*d*_−*T*_*s*_)days	–
*T*_*d*_	Time of natural death	74 ×365 days	[37]
*T*_*s*_	Time to initiate treatment after infection	Determined by *B*_*CD*4_	–
*T*_*r*_	Time of emergence of drug resistant virus	Random variable in [*T*_*s*_,*T*_*e*_]	–
*T*_*e*_	LE after infection with treatment	In [*T*_*m*_,*T*_*d*_]	–
*T*_1000_	Time to virological failure	Determined by viral loads	–
*B*_*CD*4_	Baseline CD4 counts to initiate the treatment	350 [100-450]cells/ *μ* *l*	–
*d*_*a*_	Drug adherence rate	90% [50-100%]	–

where *l,p,q* are modification factors related to corresponding parameters with *l*=0.935,*p*=3,*q*=1 as baseline values. And $\bar {k}=lk, \bar {\lambda }=l\lambda $, $\bar {\tau }_{50}=p\tau _{50}$, $\bar {\eta }=q\eta $. Note that over bar represents the same parameter but with values corresponding to with ART and/or drug resistance

Once ART is initiated for a patient, the disease progression will be changed with altered life span. Let function *τ*(*t*) denote the treatment benefit, referred as to the TBI in what follows. This function measures the integrated effects of ART on the patients’ survival so that the location parameter in the Weibull function becomes *T*_*m*_+*τ*(*t*) with the ART. Therefore, the infection rate and the viral reproduction rate under ART become

5 $$ \bar{k}(t)=\frac{\bar{k}}{W\left(T_{m}+\tau(t), \bar{\beta}_{k}, \bar{\alpha}_{k}\right)}, \bar{\lambda}(t)=\frac{\bar{\lambda}}{W\left(T_{m}+\tau(t), \bar{\beta}_{\lambda}, \bar{\alpha}_{\lambda}\right)} \ t<T_{e},  $$

where a parameter over-bar indicates the same parameter but now associated with ART, and *T*_*e*_ is the time when the patient under ART will die and this will be further explained below. In what follows, we will write $\bar {k}=lk$ and $\bar {\lambda }=l\lambda $ for a positive constant *l*.

Following the formulation of the *E*_*max*_ model [16, 29, 30], we define TBI as the saturated function that tracks the LE of the patient at any given time *t*:

6 $$ \tau(t)=\left\{\begin{array}{l} \frac{\tau_{m} \eta (t-T_{s})}{\tau_{50}+\eta(t-T_{s})}, \quad T_{s}\leq t< T_{r},\\ \frac{\tau_{m} \bar{\eta} (t-T_{s})}{\bar{\tau}_{50}+\bar{\eta}(t-T_{s})}+\tau_{T_{r}}, \quad t\geq T_{r}. \end{array}\right.  $$

**Fig. 1:**
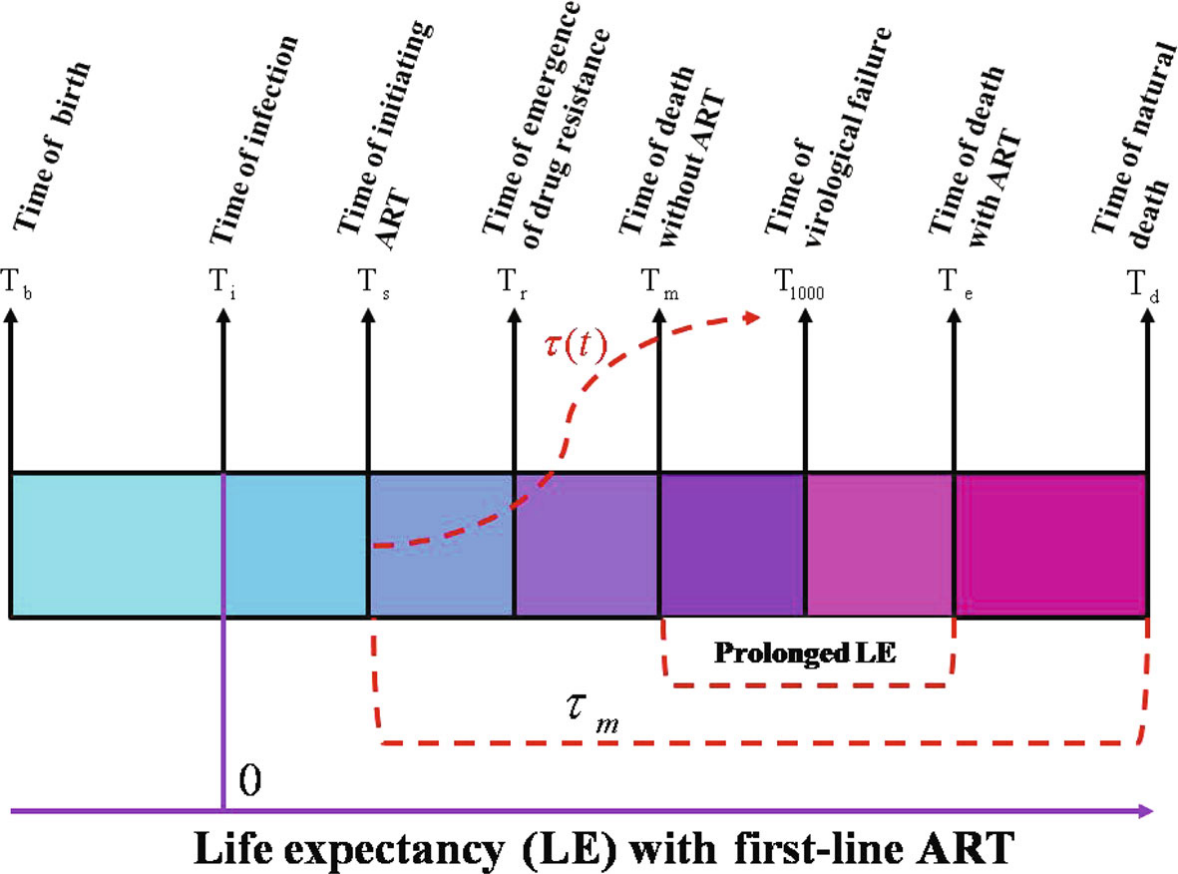
Critical points from time of infection to death. Here we set the time of infection for each patient as zero, *T*_*e*_−*T*_*m*_ represents the prolonged LE due to ART, *T*_1000_−*T*_*s*_ shows the duration of viral suppression during which the viral load is maintained below 1000 copies/ *μ* *l* under the first line ART regimens

This function links the TBI to the drug efficacy (*η*), drug sensitivity (represented by *τ*_50_), ART initiation time (*T*_*s*_) which is determined by the baseline CD4 T cell counts, time of emergence of drug-resistant virus variants (*T*_*r*_) and drug adherence (DA). We refer to Fig. 1 for various times in the entire disease progression with and without ART, where *T*_*b*_ is the time of birth, *T*_*i*_ is the time of infection with HIV, *T*_1000_ is the time of virological failure after the initiation of ART, *T*_*e*_ is the death time of the HIV infected individual with ART and *T*_*d*_ is the time of natural death of humans given no HIV infection.

Here and in what follows, *τ*_*m*_ denotes the maximum LE since ART initiating, which can be as long as the natural LE of an uninfected individual. *τ*_50_ is the duration of treatment that induces an effect equivalent to 50% of the maximal LE, quantifying agent-specific drug susceptibility. The constant $\tau _{T_{r}}$ in (6) is chosen so that the function *τ*(*t*) is continuous at *T*_*r*_, i.e.

7 $$ \tau_{T_{r}}=\tau_{m}\left[\frac{\eta (T_{r}-T_{s})}{\tau_{50}+\eta (T_{r}-T_{s})}-\frac{\bar{\eta} (T_{r}-T_{s})}{\bar{\tau}_{50}+\bar{\eta} (T_{r}-T_{s})}\right].  $$

Recall that $\bar {\eta }$ and $\bar {\tau }_{50}$ denote the reduced drug efficacy and drug sensitivity due to emergence of drug resistant variants, we have $\tau _{50}<\bar {\tau }_{50}$ and $\eta >\bar {\eta }$. Therefore, if we write $\bar {\tau }_{50}=p\tau _{50}$ and $\bar {\eta }=q\eta $, then *p*≥1 and *q*≤1. We will address the issue of **DA** and its impact on TBI in the following.

**Determination of LE with ART** It follows that both the infection rate $\bar k(t)$ and the viral production rate $\bar \lambda (t)$ become infinity at time *t* when the following life termination equation is satisfied:

8 $$ T_{m}+\tau(t)-t=0, \ T_{m}<t<\tau_{m}+T_{m}.  $$

The smallest root of the above equation is denoted by *T*_*e*_. Note that from the point view of mathematics the LE of a patient is supposed to be associated with the infinite increases of viral loads and consequently the infinity of the viral production rate. Then the smallest root of equation (8) gives the LE with ART. To calculate this smallest root, we consider the following two cases:

9 $$ T_{m}+\frac{\tau_{m} \eta (t-T_{s})}{\tau_{50}+\eta(t-T_{s})}-t=0, \quad T_{m}<t\leq T_{r}  $$

or

10 $$ T_{m}+\frac{\tau_{m} \bar{\eta} (t-T_{s})}{\bar{\tau}_{50}+\bar{\eta}(t-T_{s})}+\tau_{T_{r}}-t=0, \ \ T_{r}<t<T_{d}.  $$

Denote

11 $$ \begin{array}{rl} &B_{1}=(T_{m}\eta+\tau_{m}\eta-\tau_{50}+T_{s}\eta), \quad \ B_{2}=(T_{m}\tau_{50}-T_{m} T_{s}\eta-\tau_{m}T_{s}\eta),\\ &\bar{B}_{1}=(\bar{T}_{m}\bar{\eta}+\tau_{m}\bar{\eta}-\bar{\tau}_{50}+T_{s}\bar{\eta}), \quad \ \bar{B}_{2}=(\bar{T}_{m}\bar{\tau}_{50}-\bar{T}_{m} T_{s}\bar{\eta}-\tau_{m}T_{s}\bar{\eta}),\\ &\bar{T}_{m}=T_{m}+\tau_{T_{r}}. \end{array}  $$

Thus, solving the Eqs. (9) and (10) yields four roots

12 $$ t_{12}=\frac{B_{1}\pm \sqrt{B_{1}^{2}+4\eta B_{2}}}{2\eta},\quad \bar{t}_{12}=\frac{\bar{B}_{1}\pm \sqrt{\bar{B}_{1}^{2}+4\bar{\eta} \bar{B}_{2}}}{2\bar{\eta}}.  $$

The smallest one of real roots *t*_12_ and $\bar {t}_{12}$, lying in the interval [*T*_*m*_,*T*_*d*_], is what we want to find, and denoted by *T*_*e*_. Then *T*_*e*_−*T*_*m*_ gives the prolonged LE due to ART, indicates the integrated treatment benefits. Based on the feasibility of four roots we can provide the formula of *T*_*e*_ and one of the possible cases is discussed in the following. Note that here *T*_*d*_ is set to be equivalent to or greater than the *T*_*e*_. Due to the fact the extension of the life by ART has been increasing, we then assume that the LE of patients with ART can be as long as the average LE of individuals without infection.

Let *B*_2_≥0 and $\bar {B}_{2}\geq 0$, then we have △>0 and $\bar {\triangle }>0$, where $\triangle =B_{1}^{2}+4\eta B_{2}$ and $\bar {\triangle }=\bar {B}_{1}^{2}+4\bar {\eta } \bar {B}_{2}$, then two roots *t*_1_ and $\bar {t}_{1}$ are positive. And further, if these two roots are in their intervals, respectively, then *T*_*e*_ can be defined as

13 $$ T_{e}=\min\{t_{1}, \bar{t}_{1}\}=\min\left\{\frac{B_{1}+\sqrt{\triangle}}{2\eta}, \frac{\bar{B}_{1}+ \sqrt{\bar{\triangle}}}{2\bar{\eta}}\right\}.  $$

This formula shows how the drug efficacy (*η* or $\bar {\eta }$), sensitivity (*τ*_50_ or $\bar {\tau }_{50}$), time of emergence of drug resistant virus (*T*_*r*_) affect the prolonged LE, and consequently the disease progression. In particular, $T_{e}= (B_{1}+\sqrt {\triangle })/(2\eta)$ indicates that the drug efficacy is quite poor and the patient dies before the emergence of drug-resistant virus; while $T_{e}= (\bar {B}_{1}+ \sqrt {\bar {\triangle }})/(2\bar {\eta })$ indicates that drug-resistant variants emerge during ART when the patient is alive.

Based on above definitions and analyses, we can see that the natural HIV disease progression is defined in the interval [*T*_*i*_,*T*_*m*_], and ART prolongs the LE till *T*_*e*_. From the mathematical point of view, we can simulate the model (1) with (3–4) in the interval [*T*_*i*_,*T*_*m*_) or model (1) with (5) in the interval [*T*_*i*_,*T*_*e*_) to produce the whole disease progressions without or with ART.

**Formulation of DA** To describe the effects of DA on treatment benefit, we further extend the TBI to include the adherence rate (*d*_*a*_, the fraction of the prescribed doses of the drug which are actually taken), and also to include various patterns of randomly or regularly missed doses. Assuming that once patients take doses daily, which will contribute to the function TBI, while the TBI keeps at a day for doses missing. Let *D*_*a*_ be a set of days when doses are missed, then we have

14 $$ D_{a} \subset D_{\tau_{m}}=\left\{t, T_{s}\leq t\leq T_{e}\right\}.  $$

Denote the time intervals *T* ^[*i*]^=[*i,i*+1], with integer *i*∈[*T*_*s*_,*T*_*e*_]. The daily treatment benefit functions are as follows.

15 $$ \tau_{i}^{[T_{s}, T_{r})}(t)=\frac{\tau_{m} \eta \left(t-h_{i}^{1}-T_{s}\right)}{\tau_{50}+\eta \left(t-h_{i}^{1}-T_{s}\right)}, \ t\in T^{[i]}, \ T_{s}\leq i<T_{r}\ \text{and}\ i\notin D_{a},  $$

16 $$ \tau_{i}^{[T_{r}, T_{e}]}(t)=\frac{\tau_{m} \bar{\eta} \left(t-h_{i}^{2}-T_{s}\right)}{\bar{\tau}_{50}+\bar{\eta}\left(t-h_{i}^{2}-T_{s}\right)}+\tau_{T_{r}},\ t\in T^{[i]}, \ T_{r}\leq i\leq T_{e}\ \text{and}\ i\notin D_{a},  $$

where $h_{i}^{1}$ and $h_{i}^{2}$ represent the accumulative number of days before *i*+1 days, when drug doses are missed during treatment intervals [*T*_*s*_,*T*_*r*_) and [*T*_*r*_,*T*_*e*_], respectively. Let $\tau _{i+1}^{c_{1}}=\tau _{i}^{[T_{s}, T_{r})}(i+1)$ or $\tau _{i+1}^{c_{2}}=\tau _{i}^{[T_{r}, T_{e}]}(i+1)$, so if the dose is missed at the first treatment day (i.e. *T*_*s*_), then we have $\tau _{T_{s}}^{c_{1}}=0$.

Based on the above notations we can define TBI (*τ*(*t*)) at *i*-th interval *T* ^[*i*]^ with any pattern of drug adherence as following:

17 $$ \tau(t)=\left\{ \begin{array}{ll} \tau_{i}^{c_{1}}, & \ i\in D_{a}, \ t\in T^{[i]} \ \text{and} \ T_{s}\leq i<T_{r}, \\ \tau_{i}^{[T_{s}, T_{r})}, & \ i\in D_{\tau_{m}}\backslash D_{a}, \ \ t\in T^{[i]}\ \text{and}\ T_{s}\leq i<T_{r},\\ \tau_{i}^{c_{2}}, &\ i\in D_{a}, \ t\in T^{[i]}\ \text{and} \ T_{r}\leq i\leq T_{e}, \\ \tau_{i}^{[T_{r}, T_{e}]}, & \ i\in D_{\tau_{m}}\backslash D_{a}, \ t\in T^{[i]}\ \text{and} \ T_{r}\leq i\leq T_{e}.\\ \end{array}\right.  $$

**The data** We consider a longitudinal cohort study that recruited 464 HIV infected individuals from Aihui, Hubei and Yunnan provinces from November 2003 and the cohort has been followed until now. The ART information and clinical/lab biomarker data were collected, including viral loads every 6 months and CD4 T cell counts every 3 months. Due to the cost, viral loads for majority of patients were not tested for each follow-up, and hence data on viral loads are missing. We analyzed the data anonymously.

**Fig. 2:**
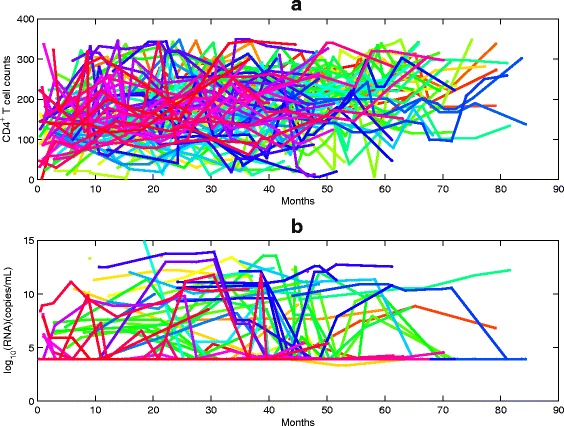
CD4 T cell counts and viral loads for 94 patients whose recorded CD4 cell counts were less than 350 cells/ *μ* *l*. **a** and **b** denote the CD4 T cell counts and viral loads, respectively

In the cohort, few patients were tested at the ART starting point, and most of patients have their first data points after a period of ART. Among these subjects, the CD4 T cell counts for 94 patients were less than 350 cells/ *μ* *l* during the ART, shown in Fig. 2. There are 149 patients whose CD4 T cell counts rebounded within interval [350, 550], 119 patients whose CD4 T cell counts rebounded within interval [550, 750], and 102 patients whose CD4 T cell counts rebounded above 750. Therefore, in order to parametrize our model from the data, to reconstruct individual patient’s disease progressions and TBI, we focused in this study on those patients for which we have sufficient data about their CD4 T cell counts and viral loads. As a result, 15 patients (listed as patient’s numbers between 1 to 15) are selected in our study, and we divide them into three groups: (G1). Four death cases (named as patients 1–4) whose CD4 counts and viral loads were tested only after a period of treatment; (G2). Three cases (patients 5–7) with ongoing first-line ART whose CD4 T cell counts and viral loads were tested at the beginning ART; (G3). Eight cases (patients 8–15) with ongoing first-line ART whose CD4 T cell counts and viral loads were tested after a period of treatment.

**The simulation method** By using the least square method and fitting the proposed model to the data together with information on first data point and/or the date of death for the death cases we estimate some model parameters which are associated with Weibull function (shape, scale and location parameters), drug sensitivity and efficacy, ART initiation time, time of emergence of drug-resistant variants and LE. Other model parameters such as the rate of supply of CD4+ T cell from precursors *s*, death rate of uninfected CD4+ T cells *d*, the baseline infection rate per virion *k* and etc are chosen from literature and listed in Table 1. Numerical simulations for the proposed model are carried out using Matlab 8, for the duration from infection (*T*_*i*_) to death (*T*_*m*_ or *T*_*e*_ with ART).

## Results

### Mimicking the entire HIV disease progression

**Fig. 3:**
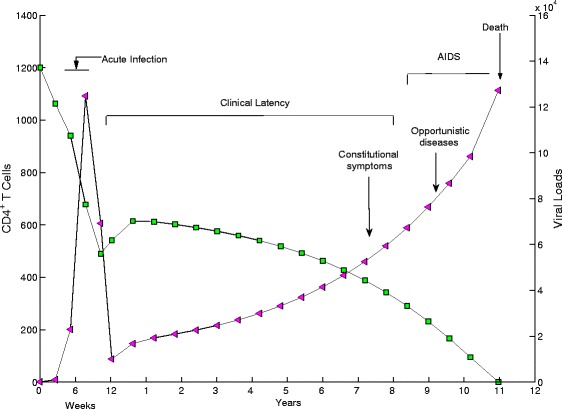
A simulated typical course of HIV infection: CD4+ T cell counts and viral load. The baseline parameters are listed in Table 1 and *T*_*m*_=11 years+12 weeks

By linking the three-parameter Weibull functions to temporal variations of infection rate and viral production rate, we obtain a non-autonomous system for the viral dynamics during the entire disease progression within a host. Our goal is to use this non-autonomous system to examine the comprehensive effect of ART on LE including the virological failure. Using the Weibull function for the temporal variation of the infection rate (*k*(*t*)) and viral production rate (*λ*(*t*)) in a classic HIV viral dynamics model, we are able to produce the viral dynamics in the entire disease progression shown in Fig. 3, faithfully capturing the observed patterns (see, for example, [31]) in the early weeks of infection, during the latency and the progression to AIDS.

**Fig. 4:**
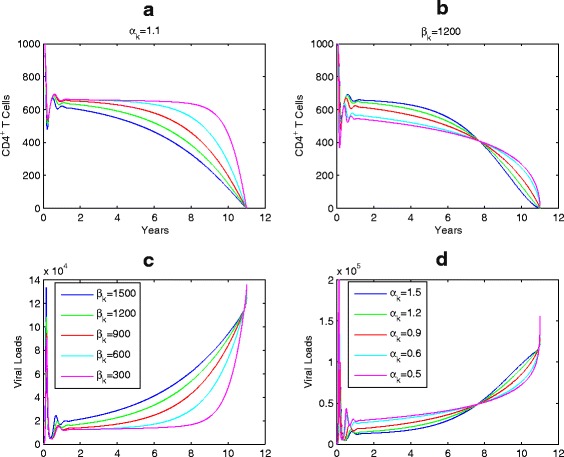
Simulations of HIV disease progressions with different scale parameters *β*_*k*_. **a**, **c**, and various shape parameters *α*_*k*_ (**b**, **d**) since infection. *T*_*m*_=11 years and other parameter values are given in Table 1

**Fig. 5:**
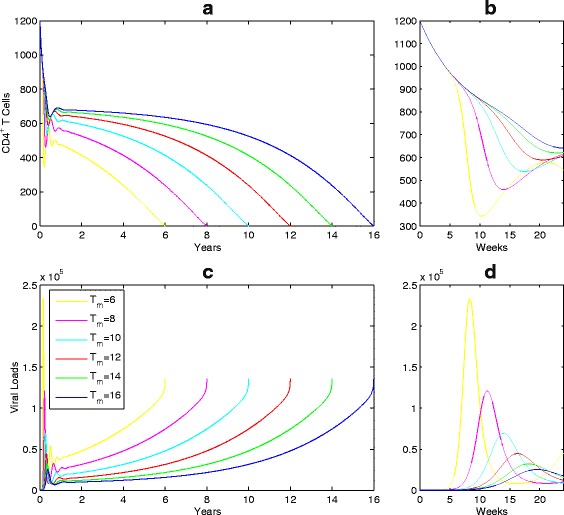
Simulations of HIV disease progressions with different life spans since infection. *T*_*m*_=6,8,10,12,14,16 years are corresponding to the curves from *yellow* to *blue*. **a**, **c** CD4 T cell counts and viral loads; (**b**, **d**). Enlargement of initial incubation infection for CD4 T cell counts and viral loads

The Weibull function, for either infection rate or viral production rate, involves three parameters: shape, scale and location parameters. Our simulations show that each of these parameters has impact on the overall patterns of disease progression. The scale parameter (*β*) governs how severe the infection is or what quasi-stationary values of the viral loads are during the latency stage (see, Fig. 4 a, c); the shape parameter (*α*) determines how fast/slow of progression to AIDS during the late stage of disease progression, as shown in Fig. 4 b, d); and the location parameter (*T*_*m*_) determines the survival time since infection (Fig. 5). This shows that different patients may have different patterns of disease progression even if they have the same life span *T*_*m*_, and that the CD4 T cell counts and viral loads during the disease progression can be highly influenced by the values of shape and scale parameters [6, 32, 33].

### Treatment benefit and LE

We apply our model to examine the integrated effect of ART on the LE of a patient. The maximum/optimal prolonged LE is given by the difference of the LE (*T*_*e*_) when the patient is on ART and the LE (*T*_*m*_) when the patient is without ART, that is, *τ*_*m*_=*T*_*e*_−*T*_*m*_ (see critical time points in Fig. 1). The actual prolonged LE defined as the *Treatment Benefit Index (TBI)* at any given time *t* since the initiation of ART (*T*_*s*_) is given by a saturated function *τ*(*t*) (6). This index summarizes the integrated effect of ART on the LE of a patient, and more importantly as will be shown in next section, this summative index can be reconstructed from the individual clinic data. The actual LE of the patient is then determined when the viral reproduction function reaches infinite, and this can be analytically calculated by finding the smallest root of a simple algebraic equation *T*_*m*_+*τ*(*t*)−*t*=0. As the TBI tracks the prolonged LE during the ART, it is natural to observe (simulations not reported here) that early initiation of ART delays disease progression and results in long LE, and further simulations show that late emergence of drug resistant variants or strong sensitivity leads to an increase in LE.

**Fig. 6:**
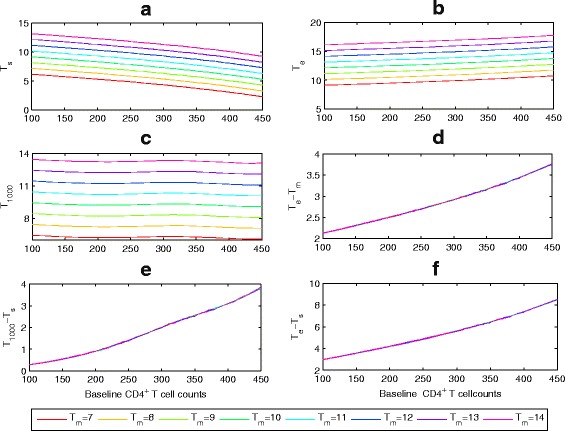
The effect of the baseline CD4 cell counts (*B*_*CD*4_) and LE when the patients is without ART (*T*_*m*_) on (**a**) the time to treatment initiation (*T*_*s*_), (**b**) LE when the patient is on ART (*T*_*e*_), (**c**) time of virological failure (*T*_1000_), (**d**) prolonged LE (*T*_*e*_−*T*_*m*_), (**e**) duration of viral suppression (*T*_1000_−*T*_*s*_) and (**f**) duration of ART. In each subplot, the parameter *T*_*m*_ is varied as 7+(*i*−1), *i*=1,2,⋯,8, where HSV color space(*red*-*cyan*-*magenta*) in Matlab is used to show the changes of parameter values and unless otherwise stated we use these notations. Here *τ*_50_=50 and baseline parameter values are listed in Table 1

The viral dynamics model when the TBI is added to *T*_*m*_ in the Weibull functions describes the disease progression during ART. This allows us to predict the virological failure time, the time when viral loads respond to 1000 copies/ *μ* *l* since starting ART. This model also allows us to examine how the baseline CD4 cell counts *B*_*CD*4_ (the CD4 cell counts to initiate ART) impacts the prolonged LE and the duration of viral suppression (*T*_1000_−*T*_*s*_). Figure 6 b, for example, shows that LE increases as the baseline CD4 cell count increases. It is interesting to observe that *T*_1000_ is not sensitive to the baseline CD4 T cell counts (Fig. 6 c), for patients with the same *T*_*m*_. On the other hand, Fig. 6 d–e shows that early initiation of ART can not only increase the prolonged LE, but also prolong the duration of viral suppression. In particular, if *B*_*CD*4_=350 copies/*μ* *l,T*_*m*_=11 years and other parameter values are fixed as Table 1, then an individual who started ART at *T*_*s*_=7.728 years after infection potentially has LE of *T*_*e*_=15.2244 years (the prolonged LE is 4.2244 years) and the duration of viral suppression is about 2.5821 years. Our simulations also illustrate that the prolonged LE or duration of viral suppression is insensitive to variation in *T*_*m*_ (Fig. 6 d–f), given the baseline CD4 T cell counts.

**Fig. 7:**
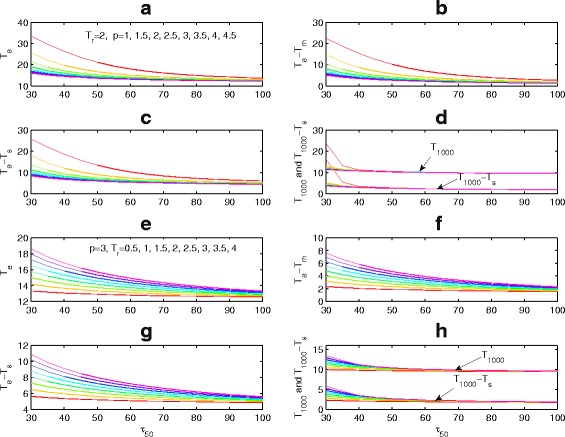
Effects of drug sensitivity parameters *τ*_50_,*p* and time of emergence of drug resistant virus (*T*_*r*_) on LE (*T*_*e*_), time of virological failure (*T*_1000_), the prolonged LE (*T*_*e*_−*T*_*m*_) and the duration of viral suppression (*T*_1000_−*T*_*s*_). **a**–**d** *τ*_50_ and *p*=1+(*i*−1)0.5; (**e**–**h**) *τ*_50_ and *T*_*r*_=*T*_*s*_+0.5+0.5(*i*−1) years, *i*=1,2,⋯,8, which is corresponding to color order from red to cyan and finally to magenta. Here *T*_*s*_=7.7228 years for *B*_*CD*4_=350 cells/ *μ* *l* and *T*_*m*_=11 years

After drug-resistant virus variants emerge, the drug sensitivity and efficacy decline [34] (with the reduction factors denoted by *p* and *q* in our study). Figure 7 b–d shows that the prolonged LE and the duration of viral suppression are shortened with declining drug sensitivity. Figure 7 f–h, on the other hand, shows that these durations are extended with the late emergence of drug resistant virus variants (large *T*_*r*_). In particular, for a patient extremely (e.g. *τ*_50_=30) or normally(e.g. *τ*_50_=80) sensitive to drugs, the prolonged LE and the duration of viral suppression increase by 1.4957 and 0.9842 or 0.4518 and 0.0012 years, respectively, with one year delay of drug resistance.

**Fig. 8:**
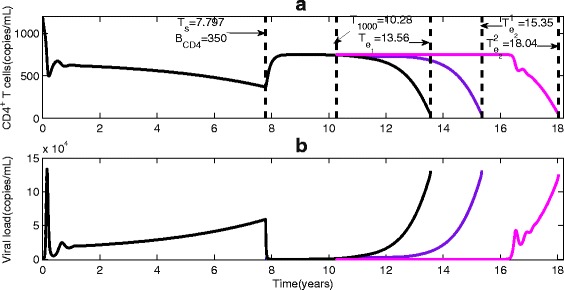
Effect of different drug efficacy and sensitiveites for the second-line regimen on HIV disease progression. **a** and **b** denote the CD4 T cell counts and viral loads, respectively. *Black curve* represents the first-line treatment only, *purple* and *pink curves* show the cases of switching from first-line regimen to the second-line regimen without and with improving drug efficacy, respectively. Parameter values for first-line regimen: $\tau _{50}=50, \bar {\tau }_{50}=150, \eta =0.95$, $\bar {\eta }=0.6, l=0.935$; Parameter values for the second-line regimen: $\tau _{50}=30, \bar {\tau }_{50}=100$, $\eta =0.95, \bar {\eta }=0.8$, *l*=0.92

Virological failure occurs when ART fails to suppress a patient’s viral loads to less than 1000 copies/ *μ* *l*, and a new treatment regimen may have to be chosen to better control the infection. We further conducted simulations of disease progression, based on the proposed models with piecewise TBI, to examine the second-line regime and its contribution to LE. If the second-line regime has superior efficacy compared with the first-line regime and patients are more sensitive to the second-line drugs than the first-line ones, it could greatly prolong LE and durably suppress viral loads, as shown in Fig. 8. This is in agreement with the finding that second-line ART in South Africa achieved durable viral suppression in three-quarters of patients [24]. However, if the second-line drugs have the relatively similar efficacy to the first-line ones, it barely suppresses viral reproduction but still can prolong the LE. This indicates that the second-line drugs should be more effective than the first-line one in order to maximize the LE of a patient.

### Effect of adherence on drug efficacy

Simulating our proposed model with function *τ*(*t*) defined (17) shows that the HIV disease progresses slowly with hight rate of adherence (not reported here). To show the effects of different DA patterns on the HIV disease progression, we consider the following two special patterns:

- *Case 1: Random pattern* Suppose does missing is a random event due to uncertainty, we randomly take the days from the interval [*T*_*s*_,*T*_*e*_] with a proportion of 1−*d*_*a*_. We run the simulations with various DA rates and again obtain that the greater DA rate the longer mean LE.
- *Case 2: Regular pattern* Let *w*_1_ (*w*_2_) be the numbers of days of drug-on (drug-off), then DA rate yields $d_{a}=\frac {w_{1}}{w_{2}+w_{1}}$. Therefore, we call the regular pattern as *nw*_1_:*nw*_2_ pattern with *n*=1,2,3,⋯, where *n* depicts the frequency of on-off pattern switching with fixed adherence rate *d*_*a*_.

A cohort study in China [34] has indicated that imperfect DA is an important factor that reduces drug efficacy and sensitivity. Thus, in order to depict this point, we assume the drug efficacy and sensitivity are functions of $h_{i}^{2}$, the dynamic accumulative days with missed doses during treatment intervals [*T*_*s*_,*T*_*e*_]. So we have the revised drug efficacy and sensitivity as the following:

18 $$ \tau_{50}^{DA}(t)\left(\bar{\tau}_{50}^{DA}(t)\right)=\tau_{50}r_{1}^{h_{i}^{2}}\left(\bar{\tau}_{50}r_{1}^{h_{i}^{2}}\right), \ \eta^{DA}(t)\left(\bar{\eta}^{DA}(t)\right)=\eta r_{2}^{h_{i}^{2}}\left(\bar{\eta } r_{2}^{h_{i}^{2}}\right), \ t\in T^{[i]},  $$

with *r*_1_>1 and close to 1, *r*_2_<1 and close to 1. Since no reliable information of how DA influences on the time of emergence of resistant virus variants, we simply do not consider the effects of DA on time of emergence of resistant strain here.

**Fig. 9:**
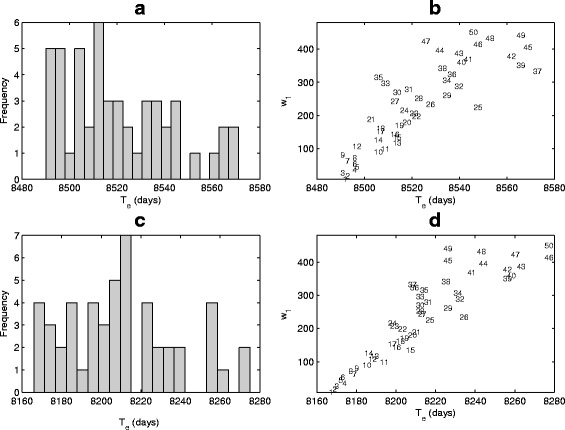
Distributions of LE (**a**, **c**) and correlation between duration of drug on (*w*_1_) and LE (*T*_*e*_) (**b**, **d**) for various regular adherence patterns at a given drug adherence rate (*d*_*a*_=90*%*). Here the ratio of the duration of drug on to drug off is 9n:1n (regular pattern), *n*=1,2,3,⋯,50. **a**–**b** *p*=1.5, *r*_1_=1 and *r*_2_=1; (**c**–**d**) *p*=1.5, *r*_1_=1.0001 and *r*_2_=0.9999. The time to initiate treatment is *T*_*s*_=7.7228 years

When the pattern of patient’s missed doses is regular, we can use the model to examine the effect of frequency of drug on-off switching on LE. Figure 9 shows the distributions of LE and correlation between duration of drug on (*w*_1_) and LE for various regular adherence patterns at a given adherence rate of 90%. Figure 9 a gives the distribution of 50 simulations with various switching frequencies and Fig. 9 b shows the positive correlation between LE (*T*_*e*_) and the duration of drug on. We conclude that a long duration of drug on (and consequently a long duration of drug off due to the fixed DA rate) could yield slightly longer LE by about 100 days. A repeat of the above with low drug efficacy and decreased sensitivity due to imperfect adherence gives a short LE (shown in Fig. 9 c, d).

In the case of randomly missed dose pattern, our simulations demonstrate that TBI decreases and disease progresses faster with less DA rate. The simulated distributions of LE again illustrate a comparative advantage of increasing adherence (not shown here). For a given adherence 60% (or 90%), the mean LE is 16.6944 (or 23.2532) years with range of 271 (177) days. Similarly, lower drug efficacy and decreased sensitivity in such a scenario give a shorter mean LE.

**Fig. 10:**
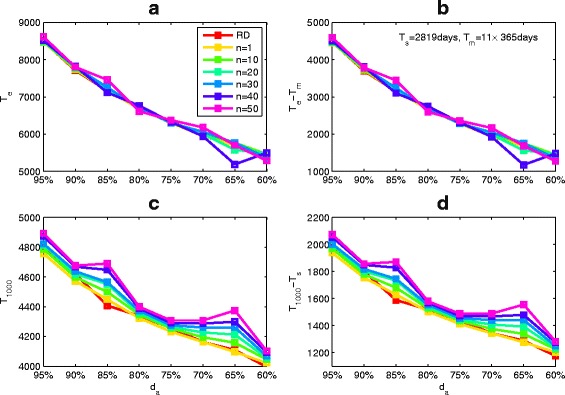
The effects of the drug adherence rate and various patterns on (**a**) the LE (*T*_*e*_), (**b**) virological failure time (*T*_1000_), (**c**) prolonged LE (*T*_*e*_−*T*_*m*_) and (**d**) the duration of viral suppression (*T*_1000_−*T*_*s*_). Here RD denotes the random pattern and regular pattern uses the *nw*_1_:*nw*_2_ to represent the ratio of duration of drug on to drug off, *n*=1,10,20,30,40,50. Some parameter values are *p*=1.5, *r*_1_=1.0001 and *r*_2_=0.9999

To examine how different DA rates and dose missing patterns with various switching frequencies influence LE and the time of virological failure, we plot variation in LE with DA rates for different patterns. Figure 10 a and b show that the LE and prolonged LE are not sensitive to adherence patterns for a given DA rate. However, timing of virological failure (*T*_1000_) and duration of viral suppression (*T*_1000_−*T*_*s*_) show great variance for different adherence patterns, especially for 85 and 65% adherence (shown in Fig. 10 c–d). This implies that frequently switching drug on and off is not beneficial to suppress viral replication, and hence results in slight shorter LE.

### A case study: reconstruction of TBI from the clinical data

**Fig. 11:**
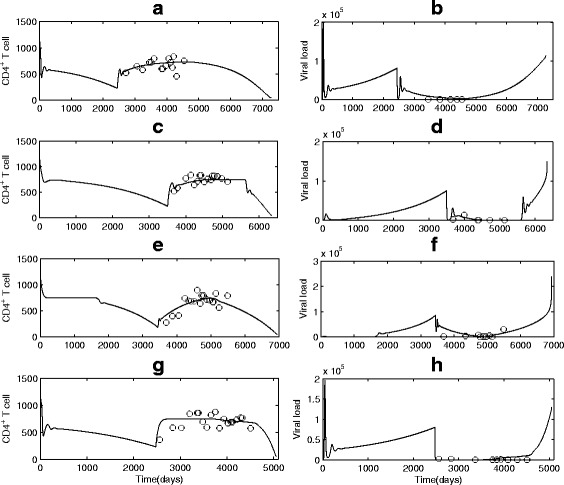
Curve fitting and parameter estimation for four continuous ART cases (G3) whose CD4 count and viral load were tested after a period treatment. **a**–**b** Patient 8; (**c**–**d**) Patient 9; (**e**–**f**) Patient 10; (**g**–**h**) Patient 11

Some model parameters are chosen from literature and other model parameters (such as shape, scale and location parameters, drug sensitivity and efficacy, ART initiation time, time of emergence of drug-resistant variants, and LE) are estimated by fitting the proposed model to the data together with information on first data point and/or the date of death for the death cases. All estimated parameter values for 15 patients are listed in the Tables 2 and 3, and the data fitting results for patient 8 to patient 11 are shown in Fig. 11 (fitting results for other patients not reported here). In summary, we observed that patients with adequate (high) CD4 response achieved relatively long prolonged LE (see patients 8 and 10 in Fig. 11), patients with poor (low) CD4 response had relatively short prolonged LE, while others whose CD4 counts barely responded to ART almost had no prolonged LE.

**Table 2:** Estimated parameter values for patients 1–7

Par.	P1	P2	P3	P4	P5	P6	P7
*β*_*λ*_	117.95	107.65	152.83	136.51	109.63	109.92	116.75
*β*_*k*_	1542.68	1614.66	1873.36	1270.91	1476.14	1471.95	1606.20
*α*_*λ*_	0.32	0.13	0.44	0.31	0.08	0.08	0.04
*α*_*k*_	0.56	0.99	0.84	1.08	1.11	1.11	1.07
*T*_*m*_	4014	3946.7	4571.1	3739.2	3995	4002.1	4019.7
*η*	0.78	0.71	0.60	0.78	0.89	0.78	0.96
*τ*_50_	117.89	39.34	383.85	30.23	52.64	55.92	31.76
*τ*_*m*_	15915	18055	4496	7574	17382	17277	20596
*q*	0.84	0.77	0.89	0.75	0.78	0.64	0.65
*p*	3.72	8.13	1.23	13.96	1.89	2.19	3.14
$\bar {\beta }_{k}$	73.35	1614.7	1869.7	12.70	1294.6	931.08	217.12
$\bar {\alpha }_{k}$	0.56	0.99	0.84	0.80	1.10	1.11	1.07
*l*	0.80	0.99	0.99	0.86	0.80	0.99	0.90
*T*_*s*_	2983.6	3681	3290.4	3358.7	3719.5	3521	3839.7
*T*_*r*_	3796.47	5070.56	3812.76	3929.77	5544.5	4424.67	4232.49
*T*_*e*_	4279.6	5116	4592.1	4038.7	4909	4615.4	6706.3
*T*_*d*_	18899.09	21736.01	7786.16	10932.50	21101.71	20798.12	24436.16
*B*_*CD*4_	200	100	200	50	–	–	–

where Par. represents parameter, and P*i* denote Patient *i*

**Table 3:** Estimated parameter values for patients 8-15

Par.	P8	P9	P10	P11	P12	P13	P14	P15
*β*_*λ*_	108.12	109.99	127.17	107.15	174.51	112.38	139.62	113.85
*β*_*k*_	1619.24	1444.27	1170.06	1626.31	1586.35	1490.67	1660.41	1576.88
*α*_*λ*_	0.03	0.13	0.22	0.04	0.13	0.06	0.05	0.08
*α*_*k*_	1.18	1.10	0.93	1.09	1.23	1.09	1.17	1.18
*T*_*m*_	3128.4	4026.7	3701.9	3118.1	3254.5	3952.6	4300.6	3220.4
*η*	0.99	0.87	0.95	0.94	0.96	0.93	0.93	0.95
*τ*_50_	30	30.09	30.71	53.27	30.22	55.79	39.33	65.58
*τ*_*m*_	21897	18753	21340	20003	21530	17541	21454	20267
*q*	0.55	0.65	0.88	0.58	0.90	0.76	0.53	0.57
*p*	4.65	4.98	3.25	2.61	3.30	2.11	3.04	2.18
$\bar {\beta }_{k}$	746.11	619.94	961.22	189.82	1164.1	1321.1	814	772.8
$\bar {\alpha }_{k}$	1.18	1.09	0.87	1.09	1.13	0.93	0.66	1.11
*l*	0.92	0.86	0.84	0.92	0.83	0.93	0.81	0.93
*T*_*s*_	2441.4	3498.6	3445.7	2472.7	2599.4	3002	3215.7	2569
*T*_*r*_	4526.31	5048.56	4863.17	2872.21	3906.73	4790.24	4418.25	4498.8
*T*_*e*_	7268.8	6339.7	6938.3	5054.7	6722.5	5473	6294.4	4708.4
*T*_*d*_	24338.38	22251.46	24785.25	22476.19	24129.89	20542.58	24669.66	22835.56
*B*_*CD*4_	200	200	150	–	200	300	300	200

where Par. represents parameter, and P*i* denote Patient *i*

**Fig. 12:**
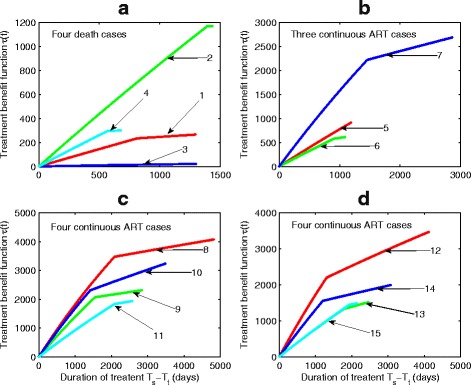
Determining the TBIs for 15 patients based on parameter estimates. **a** TBIs for four death cases; (**b**) TBIs for three patients (Group 2); (**c**–**d**) TBIs for eight patients (Group 3)

The TBI can be reconstructed based on the estimated parameter values for all 15 patients, as shown in Fig. 12. This predicts the prolonged LE for each individual patient on ongoing first-line regime. The turning point of each estimated TBI in Fig. 12 is the time of emergence of drug-resistant virus variants, after which drug efficacy and sensitivity are reduced. The TBI reported in Fig. 12 a and estimated parameters for patient 3 show that drug efficacy(*η*=0.605) and sensitivity (*τ*_50_=383.85) are both low, and this resulted in a small and slow increase of TBI over time and very insignificant increase in the prolonged LE (*T*_*e*_−*T*_*m*_=20.96 days). Other data fits (not reported here) further confirms that the first-line ART for patient 3 barely delayed the disease progression. For the patient 4 with the first recorded CD4 counts of 40 cells/ *μ* *l*, we constructed the TBI (Fig. 12 a) and calculated the prolonged LE to be 299 days (*T*_*e*_−*T*_*m*_=299.46). Patient 2 had the longest LE on the first-line ART among the four death cases, which was associated with late emergence of drug-resistant virus variants and great drug efficacy and sensitivity. The TBI for patient 8 increased with the fastest speed and latest time of emergence of drug resistant variants (Fig. 12 c). Figure 11 confirms that the disease progression of this patient was significantly delayed during ongoing first-line ART. The predicted prolonged LE for patient 8 under first-line regimen is around 11.34 years (*T*_*e*_−*T*_*m*_=4140.4 days). The TBI shown in Fig. 12 also reveals low efficacy of the first-line treatment for patients 1, 3, 4, 5, and 6. This further highlights the importance of early diagnosis of HIV infection and more effective ART regimens including second-line regimens should be considered for this cohort.

## Discussions

It is known that HIV infection typically results in a vast replication of virus during the acute phase. The viral load then becomes much lower and approaches a quasi-steady state, and finally increases significantly after the development of AIDS [6, 35]. The viral loads change overtime and behave as the ‘bathtub curve’, which shows three stages over the life time and hence is very well depicted by the proposed mixed Weibull function [23] for the temporal variability of infection rate and viral reporduction rate. Despite intensive and promising progress in HIV/AIDS viral dynamics modeling, it remains a challenge to provide approximation of the entire HIV disease progression dynamics. Here, by linking the viral reproduction rate and infection rate to the Weibull function with biologically interpretable shape, location and scale parameters, we showed that the viral dynamics model can describe a typical disease progression including acute infection, chronic latency and AIDS stage. In particular, when life expectancy is assumed to be infinity, the three-parameter Weibull function becomes unity and our proposed model reduces to the classic model of HIV dynamics [8, 19, 21].

We have also shown that our model can be used to predict the LE of an HIV infected individual and the time of virological failure. The accurate description of the entire HIV disease progression makes it possible to use this model to predict the transmission probability at different stages based on viral loads, and this is important when we consider new infections generated by a particular infected individual. Our model can also be used to determine the timing of infection for an infected individual based on individual parameters, monitored data on CD4 cell counts and viral loads, which is difficult to get. This estimation of the infection time for each infected individuals provides vital information on estimating new infections at the population level. In addition, the knowledge about the timing of infection for HIV-infected individuals in various communities enables effective contact tracing and facilitates treatment resource allocation.

Simulating the proposed model shows early initiation of ART can result in long LE (great *T*_*e*_) and prolonged LE (*T*_*e*_−*T*_*m*_), in agreement with those in previous studies [26]. Since the waiting time for the emergence of resistant genomes is substantial [36] and is incorporated in our introduced TBI, we developed a continuous (rather than an impulsive model) model of HIV dynamics with switching to describe differences of drug efficacy and sensitivity after emergence of drug resistant virus variants. Our results show that later emergence of drug resistant virus variants leads to longer (prolonged) LE and more persistent viral suppression. The estimated piecewise TBIs are increasing functions with treatment duration, with a great/low slop before/after the emergence of drug resistant virus variants. Therefore, we could estimate the time of emergence of drug-resistant variants for an infected individual, which may provide information on the time for switching to the second-line regimen without resistance testing. It is known that individualized therapy is hampered by limited availability of viral load and resistance testing, making it difficult to determine whether the remaining antiviral potency of previously used drugs outweighs their toxicity [27]. Hence, our estimation makes individualized therapy more feasible and cost-effective.

## Conclusions

The proposed novel modeling approach led us naturally to the introduction of the treatment benefit index (TBI) to summarize the integrated effect of ART in terms of prolonged LE. Moreover, this TBI can be reconstructed from clinical data with predicting the time of virological failure. Our model can be used to determine the timing of infection for an infected individual based on individual parameters, monitored data on CD4 cell counts and viral loads. Main results show that combining model predictions with monitored CD4 counts and viral loads can provide critical information about the disease progression, to assist the design of ART regimen for maximizing the treatment benefits.

## Abbreviations

AIDS: Acquired immune deficiency syndrome
ART: Antiretroviral therapy
DA: Drug adherence LE: Life expectancy
HIV: Human immunodeficiency virus
TBI: Treatment benefit index
